# Prevalence of intimate partner violence and its association with symptoms of depression; a cross-sectional study based on a female population sample in Sweden

**DOI:** 10.1186/s12889-017-4222-y

**Published:** 2017-04-20

**Authors:** Solveig Lövestad, Jesper Löve, Marjan Vaez, Gunilla Krantz

**Affiliations:** 1 Department of Community Medicine and Public Health, Sahlgrenska Academy at University of Gothenburg, Box 453, 405 30 Göteborg, Sweden; 2 Department of Clinical Neuroscience, Division of Insurance Medicine, Karolinska Institutet, SE- 171 77 Stockholm, Sweden

**Keywords:** Intimate partner violence, Depression, Population-based, Women, Sweden

## Abstract

**Background:**

Intimate Partner Violence (IPV) is the most common type of violence targeting women. IPV includes acts of physical aggression, sexual coercion, psychological abuse and controlling behaviors and these forms of violence often coexist in the same relationship. Living with IPV is associated with serious mental health outcomes such as depression and depressive symptoms. Few population based studies from Sweden have investigated the relationship between different forms of IPV and women’s depressive symptoms and even fewer used controlling behavior as an independent variable in such studies. The aim of this study was therefore to assess the prevalence of exposure to IPV in terms of controlling behavior, sexual, and physical violence and their association with self-reported symptoms of depression in a female population based sample.

**Methods:**

The cross-sectional, population based sample contained 573 women aged 18–65 years randomly selected in Sweden. Five self-reported symptoms that define depression in the Diagnostic and Statistical Manual of Mental Disorders were assessed. Physical and sexual violence were inquired about using the World Health Organization’s (WHO) Violence Against Women Instrument (VAWI), while controlling behavior was assessed with the Controlling Behavior Scale (CBS). Associations between different forms of IPV and symptoms of depression were estimated by crude and adjusted odds ratio (OR) with 95% confidence intervals (CI).

**Results:**

Bivariable associations revealed that women exposed to controlling behavior, had higher OR of depressive symptoms compared to unexposed women (OR 2.43; 95% CI 1.63–3.63). Women exposed to physical and sexual violence had also a higher OR of depressive symptoms (OR 3.78; 95% CI 1.99–7.17 and OR 5.10; 95% CI 1.74–14.91 respectively). After adjusting for socio-demographic and psychosocial covariates, all three forms of IPV showed statistically significant associations with self-reported symptoms of depression.

**Conclusions:**

A strength with this study is the analysis of controlling behavior and its association with self-reported symptoms of depression in a female population based sample. Exposure to controlling behavior, physical and sexual violence by an intimate partner were clearly associated with women’s self-reported symptoms of depression.

## Background

Intimate Partner Violence (IPV) perpetrated by a current or former partner is the most common type of violence targeting women [1] and continues to be a gross violation of women’s human rights as well as a major public health problem globally [2]. The World Health Organization (WHO) defines IPV as: *‘any behavior within an intimate relationship that causes physical, sexual or psychological harm, including acts of physical aggression, sexual coercion, psychological abuse and controlling behaviors’* [3]. The term ‘controlling behavior’ includes acts that restrict a woman’s mobility or her access to relatives and friends while ‘psychological abuse’ refers to threats, insults, and acts that belittle or humiliate the partner [4]. However, in most studies controlling behavior is viewed as a form of psychological violence [5] and this is therefore inquired about and analyzed as a unitary construct [6–8]. The importance of distinguishing controlling behavior from other acts of psychological violence has repeatedly been emphasized by Johnson [9], as a form of IPV that has devastating health and social consequences both in itself and in combination with physical and/or sexual violence.

A recently published report from the 28 Member States of the European Union (EU) showed that over one in five women in the EU had experienced physical and sexual violence from either a current or former intimate partner and 35% had experienced controlling behavior [5]. In one of our earlier studies performed in Sweden, 8% of the women reported exposure to physical IPV during the past year while 3% reported exposure to sexual IPV [10]. Another study performed in Sweden showed that almost 2% of the female respondents had experienced systematic and repeated acts of controlling behavior during past year prior to the survey [11].

Previous research has shown that various forms of IPV generally coexist in the same relationship [12–14]. However, some studies suggest that psychological abuse, including controlling behavior, is far more frequent than other forms of IPV [14, 15] and that most women exposed to physical IPV, also are exposed to some form of psychological abuse [16, 17]. A study performed in Sweden for instance, showed that four out of ten women who reported exposure to jealousy from their partner, also reported exposure to physical and sexual violence [18].

In addition to physical injury, it has repeatedly been demonstrated that IPV is associated with mental health problems, including depression and depressive symptoms [19–22]. Depression is the most frequent mental health problem among women and is twice as common in women as in men [23]. Symptoms and severity of depression varies largely and may include self-reported measures as well as diagnoses based on the Diagnostic and Statistical Manual (DSM) [24]. Apart from individual suffering, depression leads to high societal and economic burden in terms of disability, sick-leave and health care costs [25].

Many studies have reported on IPV as an overarching construct, i.e. not separating the various forms of IPV when analyzing its impact on depressive symptoms [21, 26–28]. Despite the fact that psychological abuse is more prevalent than other forms of IPV, most studies have focused solely on physical and sexual IPV as exposure variables [22]. Johnson and colleagues [29] for example, found in their longitudinal study that exposure to physical IPV was associated with depressive symptoms. Likewise, sexual IPV has been identified as an important and independent risk factor for later depression in women [22, 30]. Furthermore, studies that included psychological abuse as a separate and independent variable have with few exceptions, demonstrated a consistent relationship with depression [6, 15, 31]. Additional factors known to be associated with depressive symptoms are having poor social support [21], younger age [32], being single [21], being unemployed [33] and having witnessed inter-parental IPV [34].

There is limited research on the association between different forms of IPV and women’s depressive symptoms in population based samples in Sweden. Earlier studies on this matter were mainly conducted on clinical samples [35–40] or on specific target groups, e.g., Thai women, or women from shelters [41, 42], i.e. studies not representative of the general female population. Many of the earlier studies addressed the association between violence victimization and mental health without asking the respondent to specify whether the perpetrator had been an acquaintance, a stranger, a family member or an intimate partner, thus limiting the possibility to distinguish between IPV and other forms of violence [43–45]. Since IPV is the most common type of violence against women [1], it is important to provide information about its forms and consequences in a general population-based sample of women in Sweden as preventive measures and treatment differ considerably depending on who is the perpetrator. To the best of our knowledge, few if any studies performed in Sweden include controlling behavior as an independent variable in studies on exposure to IPV and its association with symptoms of depression among women.

The aim of this study was therefore to assess the prevalence of exposure to IPV in terms of controlling behavior, sexual and physical violence and its association with self-reported symptoms of depression in a female population based sample in Sweden.

## Methods

### Design and sample

The present cross-sectional study was based on survey data extracted from a larger programme on exposure and perpetration of IPV among men and women in Sweden [46]. Between January and March 2009, a postal survey administered through Statistics Sweden was sent out to a random national sample of 1006 women and 1009 men, aged 18–65 years residing in Sweden. A short letter with information about the study and possibility to deny further participation was sent out to all selected individuals one week before the questionnaire was sent out. This was done as a security measure, to avoid aggravating possible violence exposure. The respondents were guaranteed full anonymity. For further information and/or assistance in case of being exposed to IPV, respondents were given contact details to a general practitioner, a psychologist and a contact person at Statistics Sweden. The study included two reminders in order to minimize the drop-out rate.

A total of 624 women (62.0%) and 458 men (45.5%) returned the questionnaire. Respondents with missing values (*n* = 110) on all the violence items were excluded, leaving a final sample of 972 men and women. The current study is based on 573 women who formed the final sample. Earlier drop-out analysis on this sample found that women born outside Sweden, of younger age, unmarried and with a low annual income were over-represented in the group of non-responders [46].

### Variables

#### Outcome variables

Self-reported symptoms of depression were assessed using five indicators of depression as defined in the Diagnostic and Statistical Manual of Mental Disorders, Fourth Edition (DSM- IV) [47]. Indicators of depressive symptoms according to the DSM-IV include feelings of sadness and discouragement, initial insomnia, decreased energy and tiredness as well as impaired ability to concentrate [47]. Symptoms of depression further encompasses suicidal ideation and attempts [47]. The respondents were asked if they had experienced any of the following five symptoms during the past 12 months: ‘*Tiredness/ fatigue’, ‘difficulty falling asleep’, ‘trouble concentrating’, ‘feeling down/ low’* and *‘suicidal thoughts’*. A four point scale (‘*Almost every day’, ‘Once a week’, ‘Once a month’ and ‘Almost never or never’)* was used to indicate the frequency of the various depressive related symptoms experienced in the past year. The response options ‘*almost every day’* and *‘once a week’* were merged and considered as exposed. For the bivariable and multivariable analysis, the five items were summarized and dichotomized into the exposure category, defined as two or more out of five symptoms and the reference category of having one or no such experience of depressive symptoms.

#### Exposure variables

The different forms of IPV were analyzed as primary exposure variables. Being exposed to IPV, as defined in this study, was self- reported experience of exposure to controlling behavior, physical and sexual violence during past 12 months and perpetrated by a current or former partner including spouses, common-law partners or boy/ girlfriends within opposite and same-sex relationships. The WHO Violence Against Women Instrument (VAWI) was used to assess *physical* (6 items) and *sexual* (3 items) *violence* [48] perpetrated by an intimate partner (Table 1). VAWI demonstrated good construct validity and internal reliability in one of our earlier studies performed on this sample [49]. Questions on exposure to controlling behavior were measured through the subscale *‘isolating control’* (5 items) (Table 1) from the Controlling Behavior Scale (CBS) developed by Graham-Kevan and Archer [50]. The original 5-point response format ranging from 0 (never) to 4 (always) [50] was modified into frequency questions. For each question on physical, sexual violence and controlling behavior, respondents were asked how often they had experienced a specific act during the past 12 months. The response options were ‘0 times’, ‘1 time’, ‘2 times’, ‘3–5 times’ or ‘> 5 times’. For each item, exposure was considered as present if the respondent had experienced violent behavior ‘1 to >5 times’ during the past year. Due to few cases in each of the frequency categories the cumulative number was used in the further analyses. For further bivariable and multivariable analysis, the three forms of violence were analyzed separately. Items included in each of the three subscales (‘isolating control’, physical- and sexual violence), were summarized and dichotomized into a binary variable where exposure to violent behavior was defined as having reported exposure to at least one of the items in each subscale as opposed to experiencing no such violence.

**Table 1:** Exposure to intimate partner violence presented as past year frequency (n) and percentage (%) of the total population. *N* = 573 women

Intimate partner violence	Exposure during past 12 months	
	%	*n*
Controlling behavior		
Tried to restrict time spent with my family and friends	4.9	28
Wanted to know where I went and who I spoke to when not together	17.6	101
Tried to restrict my activities outside the relationship	6.4	37
Felt suspicious and jealous of me	12.6	72
Tried to control my activities	6.6	38
*Exposed to ≥ 1 item of controlling behavior*	*25.0*	*143*
Physical violence		
Pushed or shoved me	6.6	38
Thrown something that could have hurt me	1.6	9
Hit me with the fist or with some other object	1.2	7
Kicked and dragged me and beaten me up	0.5	3
Choked or burnt me on purpose	0.5	3
Hurt me with a knife, a gun or some other weapon	0.2	1
*Exposed to ≥ 1 item of physical violence scale*	*7.5*	*43*
Sexual violence		
Demanded to have sex with me even though I did not want to	2.4	14
Forced me to have sex against my will by using physical strength	0.3	2
Forced me to perform sexual acts that I experienced as degrading and/ or humiliating	0.3	2
*Exposed to ≥ 1 item of sexual violence scale*	*2.8*	*16*

#### Covariates

For descriptive analysis *Age* was divided into five age intervals; 18–25, 26–35, 36–45, 46–55 and 56–65 years and for further analysis categorized into 18–25 years and 26–65 years with the latter as reference category. *Civil status* was categorized into three groups: (1) single, widowed, divorced; (2) boyfriend, girlfriend; (3) married, cohabitant, registered partnership, and later dichotomized by merging the two former categories. *Educational level* was categorized into three groups (university, high school and elementary school). The categories ‘university/high school’ were combined and used as the reference category. *Employment status* was grouped into seven categories: (1) employed; (2) student; (3) early retirement pension/retired; (4) sick leave; (5) parental leave or leave of absence; (6) unemployed; (7) home-worker/taking care of the household. The categories were dichotomized, using ‘employment’ and ‘parental leave or leave of absence’ as the reference group.

The measure *‘Access to social support’* has been used in the Swedish Level of living surveys (LNU) [51] and was constructed out of the question: ‘One sometimes needs help and support from someone. Do you have any relative or friend who helps out…’ followed by four subsequent questions: (1) if you become ill?; (2) if you want company?; (3) if you need to talk to someone about personal problems?; (4) if you need a loan of 15,000 Swedish crowns (at that time approx. 2200 USD)? Answering ‘yes’ to all four questions was categorized as ‘access to good social support’ whereas answering ‘no’ or ‘unsure’ to any of the four items was considered as ‘poor social support‘.

*Grown up and witnessed IPV as a child* was constructed out of two introductory questions followed by more detailed questions. The first question; ‘Have you grown up in a home where there was physical, psychological or sexual violence between your parents or the adults you lived with?’ was followed by the response options ‘no’; ‘yes’ and ‘unsure’. If the answer was ‘yes’, the respondent was asked to indicate which type of violence (physical, psychological and/or sexual violence). The second question asked whether the respondent had witnessed (heard or seen) the violence or not. The response options were ‘no’; ‘yes’ or ‘unsure’. A binary variable was constructed in which women responding ‘yes’ to both introductory questions were considered to have grown up and witnessed violence and were thus defined as ‘exposed’.

### Statistical analyses

Analyses were computed using the statistical program SPSS, version 17 and 20. Descriptive statistics using prevalence (%) and frequency (n) were used for prevalence rates. The overlap between physical, sexual violence and controlling behavior during past 12 months was illustrated by a Venn-diagram. Bivariable and multivariable analyses were performed producing crude and adjusted odds ratios (OR) with 95% Confidence Intervals (CI) in order to analyze associations between different forms of IPV, covariates and symptoms of depression.

Exposure variables that showed statistically significant associations with symptoms of depression in the crude analyses were entered one-by-one in a stepwise manner into the hierarchical logistic regression analysis. Models were created with the composite measurement of symptoms of depression and each form of IPV. Despite non significance in the bivariable analyses, *age* was included in the multivariable analyses because of its known association with both depression [32] and IPV [5]. The first model was adjusted for *age, civil status* and *employment status* and the second model was adjusted for *age, civil status*, *employment status* and *access to social support.* The third model was adjusted for *age, civil status, employment status, access to social support and grown up with and witnessed IPV as a child*.

#### Internal reliability

To assess the internal consistency of the items measuring self- reported depressive symptoms, Cronbach’s α was computed to 0.76.

### Ethical considerations

The current study was conducted in accordance with the WHO’s ethical and safety recommendations for research on IPV [52]. Approval was provided from the Regional Ethics Review Board in Gothenburg (Dnr: 527–08).

## Results

### Descriptive statistics

The sample consisted of 573 women aged 18 to 65 years, with an average age of 42.7 years (Standard Deviation =13.01). The majority of women (73.3%) were married, cohabiting or in a registered partnership (Table 2) and seven (1.2%) women reported having a same-sex relationship. Of the sample, 47.1% of the women had a university degree and further 69.7% were employed. Of the respondents, 7.7% had grown up with- and witnessed IPV as a child.

**Table 2:** Socio - demographic and psychosocial characteristics. *N* = 573 women

Characteristics	Percent%	Number
Age groups		
18–25	10.5	60
26–35	23.6	135
36–45	22.5	129
46–55	20.9	120
56–65	22.5	129
Civil status		
Single/ widowed/ divorced	14.8	85
Boyfriend/ girlfriend	11.2	64
Married/ cohabitant/ registered partnership	73.3	420
Duration of present relationship		
> 10 years	50.3	288
4-10 years	20.1	115
≤ 3 years	14.3	82
Country of birth		
Sweden	90.6	519
Other Nordic country	2.6	15
Other European country	3.1	18
Country outside of Europe	3.7	21
Educational level		
University	47.1	270
High school (10–12 years)	36.8	211
Elementary school (≤ 9 years)	15.9	91
Employment status		
Employed	69.7	396
Student	6.2	35
Early retirement pension/Retired	8.2	47
Sick leave (more than 3 months)	1.4	8
Parental leave or leave of absence	6.2	35
Unemployed	4.0	23
Home-worker, taking care of the household & other	4.2	24
Grown up with- and witnessed IPV		
No/ Unsure	91.3	523
Yes	7.7	44
Access to social support		
Good	62.8	360
Poor	34.4	197

### Prevalence, frequency and co-occurrence of controlling behavior, physical and sexual violence

**Fig. 1:**
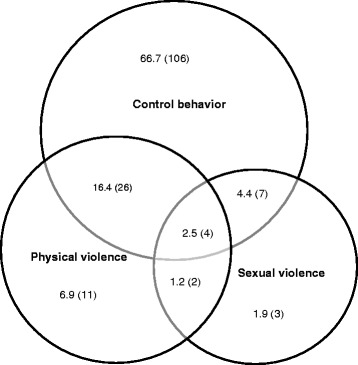
Overlap of exposure to physical and sexual violence and controlling behavior (*N* = 159). Presented as past year frequency (n) and percentage of women exposed to any type of violence

As shown in Table 1, the most common form of violence was controlling behavior (25.0%) during the past 12 months, followed by physical violence (7.5%) and sexual violence (2.8%). The most prevalent acts of controlling behavior were ‘*Wanted to know where I went and who I spoke to…*’ and ‘*My partner felt suspicious and jealous of me*’ with prevalence rates of 17.6% and 12.6% respectively*.* In total 28% (*n* = 159) of the women were exposed to at least one type of violence during the past 12 months (Fig. 1). Among the exposed women, 16.4% were exposed to controlling behavior and physical violence while 4.4% of the women were subjected to both controlling acts and sexual violence. Experience of all three forms of violence accounted for 2.5% (Fig. 1) of the respondents.

### Prevalence and frequency of self-reported symptoms of depression

Of the total sample, 31.6% of the women reported experience of at least two out of five symptoms of depression almost every day or once a week during the past 12 months (Table 3). Among the women 45.7% reported experiencing noticeable fatigue and tiredness every day or once a week while 29.7% reported difficulties falling asleep and further 18.3% had experienced difficulties in concentrating during the past 12 months. Among the respondents, 0.7% (*n* = 4) had experienced suicidal thoughts almost every day or once a week throughout the year previous to the study.

**Table 3:** Self-reported symptoms of depression presented as past year frequency (n) and percentage (%) of the total population. *N* = 573 Women

Self-reported symptoms of depression past 12 months	Almost every day	Once a week	Once a month	Almost never/ Never	Exposed almost every day/once a week
	% (n)	% (n)	% (n)	% (n)	% (n)
Fatigue/ tiredness	15.5 (89)	30.2 (173)	33.3 (190)	17.1 (98)	45.7 (262)
Difficulty falling asleep	8.7 (50)	20.9 (120)	26.7 (153)	40.5 (232)	29.7 (170)
Trouble concentrating	6.1 (35)	12.2 (70)	25.0 (143)	50.1 (287)	18.3 (105)
Feeling down/ low	6.3 (36)	11.3 (65)	31.2 (179)	44.9 (257)	17.6 (101)
Suicidal thoughts	0.5 (3)	0.2 (1)	3.5 (20)	89.0 (510)	0.7 (4)
Exposed to number of depression related symptoms (almost every day/ once a week) past 12 months					
Number of depression related symptoms					Exposed almost every day/once a week
					% (n)
1 symptom					23.7 (136)
2 symptoms					15.4 (88)
3 symptoms					7.9 (45)
4 symptoms					7.9 (45)
5 symptoms					0.5 (3)
At least 2 out of 5 symptoms					31.6 (181)

### Crude associations between IPV and self-reported symptoms of depression

Among women exposed to controlling behavior, 38.5% reported at least two out of five symptoms of depression almost every day or once a week (Table 4) and the odds for depressive symptoms was 2.43 with 95% CI of 1.63–3.63 as compared to the non-exposed. Women exposed to controlling behavior alone (without physical or sexual violence), were more likely to report depressive symptoms (*n* = 43), compared to women without such experience (OR 1.72; 1.10–2.67) (not shown in table). Further, women exposed to physical violence and those exposed to sexual violence had higher odds of reporting depressive symptoms (OR 3.78; 1.99–7.17 and OR 5.10; 1.74–14.91 respectively) compared to women with no experience of such violence. Likewise, being single/divorced/widowed, being unemployed, having poor social support and having grown up with- and witnessed IPV as a child, all demonstrated significant crude associations with self-reported symptoms of depression (Table 4).

**Table 4:** Bivariable associations between exposure to partner violence, covariates and self-reported symptoms of depression. Presented as past year prevalence (*n*), percentage (%) and crude odds ratio (OR) and 95% confidence intervals (95% CI). *N* = 573 women

Explanatory variables and potential confounders	Self-reported symptoms of depression past 12 months	
	Experienced ≥ 2 out of 5 symptoms	Crude OR (95% CI)
	% (*n*)	
Controlling behavior past 12 months		
Unexposed	61.5 (104)	1
Exposed (**≥**1 of the items)	38.5 (65)	2.43 (1.63–3.63)
Physical violence past 12 months		
Unexposed	84.6 (143)	1
Exposed (**≥**1 of the items)	15.4 (26)	3.78 (1.99–7.17)
Sexual violence past 12 months		
Unexposed	93.5 (158)	1
Exposed (**≥**1 of the items)	6.5 (11)	5.10 (1.74–14.91)
Age groups		
26–65	86.7 (157)	1
18–25	13.3 (24)	1.49 (0.86–2.58)
Civil status		
Married, cohabitant, registered partnership, boy- or girlfriend	77.2 (139)	1
Single, widowed, divorced	22.8 (41)	1.46 (1.53–3.96)
Educational level		
University/ High school	83.9 (151)	1
Elementary school	16.1 (29)	1.00 (0.62–1.62)
Employment status		
Employed, parental leave, leave of absence	64.1 (116)	1
Student, unemployed, sick-leave, early retirement /retired, home-worker, other	35.9 (65)	2.45 (1.64–3.64)
Social support		
Good	50.3 (88)	1
Poor	49.7 (87)	2.45 (1.70–3.56)
Grown up with- and witnessed IPV		
No/ Unsure	85.4 (152)	1
Witnessed violence	14.6 (26)	3.92 (2.05–7.52)

### Adjusted associations between IPV and self-reported symptoms of depression

After adjusting for *age, civil status, employment status, access to social support* and *grown up with-and witnessed IPV as a child*, all three forms of IPV still showed statistical significance with self-reported symptoms of depression (Model 3, Table 5). Women exposed to controlling behavior during past 12 months were more likely to report symptoms of depression (OR 2.43; 1.56–3.79) compared to unexposed women. Likewise, women exposed to physical and sexual violence, had higher odds to report such symptoms (OR 3.06; 1.50–6.24 and OR 4.67; 1.35–16.18 respectively) compared to the reference categories.

**Table 5:** Associations between partner violence exposure, covariates and symptoms of depression. Presented as adjusted Odds Ratios and 95% confidence intervals (95% CI). *N* = 573 women

Explanatory variables and confounders	Model 1	Model 2	Model 3
Controlling behavior past 12 months			
*(Unexposed* vs. *Exposed)*	2.44 (1.61–3.71)	2.27 (1.48–3.50)	2.43 (1.56–3.79)
Age			
*(26–65 years* vs *18–25)*	0.85 (0.46–1.59)	0.90 (0.48–1.68)	0.83 (0.44–1.60)
Civil status			
*(Married, cohabitant* vs. *single, divorced)*	2.51 (1.43–4.41)	2.62 (1.48–4.64)	2.75 (1.53–4.95)
Employment status			
(*Employed* vs. *not employed)*	2.19 (1.42–3.40)	1.94 (1.23–3.06)	1.97 (1.23–3.14)
Social support			
*(Good* vs. *poor)*		1.90 (1.27–2.86)	1.88 (1.24–2.85)
Grown up with- and witnessed IPV			
*(No/ unsure* vs. *witnessed IPV)*			3.92 (1.72–8.91)
Physical violence past 12 months			
*(Unexposed* vs. *Exposed)*	3.60 (1.84–7.04)	3.01 (1.52–5.98)	3.06 (1.50–6.24)
Age			
*(26–65 years* vs *18–25)*	0.86 (0.46–1.60)	0.86 (0.46–1.62)	0.81 (0.42–1.54)
Civil status			
*(Married, cohabitant* vs. *single, divorced)*	2.61 (1.46–4.65)	2.73 (1.52–4.92)	2.80 (1.53–5.13)
Employment status			
(*Employed* vs. *not employed)*	2.26 (1.46–3.50)	2.03 (1.28–3.21)	2.06 (1.30–3.29)
Social support			
*(Good* vs. *poor)*		1.92 (1.28–2.88)	1.92 (1.27–2.90)
Grown up with- and witnessed IPV			
*(No/ unsure* vs. *witnessed IPV)*			3.56 (1.58–8.02)
S			
*(Unexposed* vs. *Exposed)*	5.02 (1.52–16.57)	4.47 (1.31–15.26)	4.67 (1.35–16.18)
Age			
*(26–65 years* vs *18–25)*	0.93 (0.50–1.73)	0.93 (0.49–1.75)	0.85 (0.44–1.64)
Civil status			
*(Married, cohabitant* vs. *single, divorced)*	2.62 (1.48–4.64)	2.71 (1.51–4.88)	2.77 (1.52–5.01)
Employment status			
(*Employed* vs. *not employed)*	2.13 (1.38–3.30)	1.91 (1.21–3.01)	1.96 (1.23–3.12)
Social support			
*(Good* vs. *poor)*		2.09 (1.40–3.12)	2.08 (1.38–3.13)
Grown up with- and witnessed IPV			
*(No/ unsure* vs. *witnessed IPV)*			3.53 (1.57–7.96)

It was also of interest to note that of the covariates analyzed in the multivariable analyses, all variables (*single/divorced, unemployed, poor access to social support, grown up with-and witnessed IPV*) except *low age* were associated with self-reported symptoms of depressive disorders during past 12 months (Table 5).

## Discussion

This is one of few population-based studies performed in Sweden that investigated the association between different forms of IPV, i.e. controlling behavior, physical and sexual violence, and self- reported symptoms of depression during past 12 months.

### Exposure to IPV and its association with self-reported symptoms of depression

Consistent with earlier findings, we found that women exposed to controlling behavior, physical- and sexual violence by an intimate partner were more likely to report symptoms of depression [16, 20, 22] compared to women not exposed. A study performed on middle-aged women in Australia for instance, found that of those ‘sometimes’ or ‘often’ experiencing symptoms of depression, 20% and 28% respectively reported exposure to IPV [27]. Traumatic and psychological stress reactions are considered to be the core mechanisms that explain why IPV may cause subsequent depression in women [2]. Recent biomedical findings suggest that sustained psychological stress due to social threat or rejection may up-regulate proinflammatory cytokine activity which can alter the activity of neurons and neural systems that regulate cognition, mood and behavior [53]. These changes could lead to symptoms of depression through disturbances in sleep- and wake activity, decreased interest in feeding and socializing with others [53]. Likewise, it is widely known that among people with a history of depression, previous exposure to stressful life events is more common compared to those with no such history [20, 54].

In line with previous research [55, 56] findings from the current study revealed that women exposed to controlling behavior alone, i.e. without physical or sexual violence, during the past 12 months had higher odds to report depressive-related symptoms compared to unexposed women. This supports the repeatedly confirmed findings in earlier literature that psychological abuse, including controlling behavior is as detrimental to women’s mental health as other forms of IPV [16, 57]. Controlling behavior is used by the perpetrator in order to obtain obedience and dependency by depriving the partner from a range of important aspects in everyday life such as access to support systems, economic resources, social life and the right to employment and wage-earning [58]. Some authors suggest that within opposite-sex relationships, controlling behavior is perpetrated primarily by male partners [9, 58] and therefore frequently found to be experienced by women from agency samples [9]. However, our findings together with previous research on population based samples indicate that controlling behavior is experienced also among women in population-based samples [5, 18, 56]. By third-parties, controlling behavior is often perceived as less harmful and more acceptable than physical violence [59]. Controlling behavior however, has shown to be at least as harmful to women’s mental health as physical and sexual violence, even in cases were no physical violence is present [31]. It has likewise been demonstrated that when psychological violence includes power and control tactics, the associations between psychological violence and depressive symptoms further increase [56].

In accordance with previous studies, we found that controlling behavior and physical violence were those forms of violence that overlapped to the largest extent [16]. In our sample however, we found that 2.5% of the women were exposed to all three forms of violence which is a smaller proportion compared to the findings in a study by Thompson and his colleagues [14], where 30% of the women had experienced multiple forms of violence. Due to small sample size, further analyses of associations between those exposed to physical or sexual violence only (*n* = 11 and *n* = 3 respectively) and self-reported symptoms of depression was not possible. It is further important to note that since we did not adjust for each type of IPV exposure when analyzing associations between IPV and symptoms of depression, it is likely that there might be an interaction between- or an additive effect of the different types of IPV and its association with symptoms of depression.

We found that poor social support was independently associated with self-reported symptoms of depression, which is congruent with earlier research [21, 57]. Good social support has been shown to predict recovery from depressive symptoms among women exposed to IPV [57], whereas an accumulation of poor social support at a younger age is independently associated with internalizing symptoms later in life [60]. This suggests that improving women’s access to social support would mitigate symptoms of depression in women exposed to IPV.

Consistent with previous research, we also found that women being single, widowed or divorced [21], being unemployed [33] and having grown up with- and witnessed inter-parental violence [34] contributed to self-reported symptoms of depression. Previous research has shown that unemployed women are at increased risk for depressive symptoms compared to employed women, and the risk increases even more when women are exposed to IPV [33]. This together with our findings suggests that social support and restricted occupational opportunities may interact with the relationship between IPV and mental health problems.

### Methodological considerations

A strength with this study is that few if any earlier studies performed in Sweden, have analyzed the associations between controlling behavior and symptoms of depression in a female population based sample. However, some limitations should be considered when interpreting the results from this study. We are aware that women’s exposure to psychological violence is not limited to acts of controlling behavior. It also includes other forms of psychological violence such as threats, intimidation and belittling [5] which substantially contribute to women’s symptoms of depression [7]. However, our aim was to specifically address the association between controlling behavior and symptoms of depression since this form of psychological abuse is qualitatively different compared to other forms of violence in that it restricts women’s basic autonomy, liberty and freedom [58] and may be more consequential for women’s mental health over time compared to other forms of verbal/ psychological abuse [6].

Questions about witnessing inter-parental violence as a child, IPV exposure and depressive symptoms were all based on retrospective self-reporting and may therefore have been subjected to recall bias, as well as to systematic non-disclosure. This in turn may have led to an underestimation of reported associations. Past emotions and behaviors are difficult to recall in an accurate way and historical responses of psychiatric symptoms might be biased by the respondents’ current mental health status [61]. Further, obtaining reliable data on IPV is a difficult and complex task since it cannot be directly observed and in addition, it is surrounded by taboos, feelings of guilt, fear and shame [62].

Consistent with earlier findings in research on IPV [11], women born outside Sweden, of younger age, unmarried and with a lower annual income were somewhat underrepresented in our study population. Rates of IPV exposure are found to be higher in these groups [3, 36] hence the prevalence of exposure to IPV in this study might be underestimated and consequently have led to an underestimation of its associations with depressive related symptoms. According to previous research, symptoms of depression might also be underestimated in our study, since sociodemographic factors such as living alone, low income and younger age are related both to mental disorders and non- participation rates in studies [63]. Since this study was a cross sectional survey, we did not have any information on the temporal relationship between the onset and end of the reported violence or the depressive symptoms. This precludes the determination of causality, i.e. IPV may cause depression but depression may cause IPV. However, findings from longitudinal studies suggest a consistent and independent causal link between exposure to IPV and depressive symptoms [21, 64].

Due to few cases in each of the frequency categories, the included frequency levels could not be investigated as single entities. Co-occurrence of different forms of IPV and its association with symptoms of depression gave small sample sizes and no further analyses were performed.

Another limitation is that we did not use any recognized and validated instrument to measure self-reported symptoms of depression. However, from a clinical point of view, many women present at the health care center with symptoms of depression and therefore it is important to make clinicians aware of the fact that such symptoms may mirror depression that eventually may be associated with exposure to IPV.

## Conclusions

A strength with this study is the analysis of controlling behavior and its association with self-reported symptoms of depression. Findings from the current study supports previous research in that women’s exposure to controlling behavior, as well as physical and sexual violence perpetrated by a male intimate partner is clearly associated with symptoms of depression.

## Abbreviations

CI: Confidence Interval
CSB: Controlling Behavior Scale
DSM-IV: Statistical Manual of Mental Disorders, Fourth Edition
IPV: Intimate Partner Violence
LNU: Swedish Level of Living Survey
OR: Odds Ratio
WHO: World Health Organization
